# Severe iron-deficiency anemia and recurrent mucosal bleeding revealing Glanzmann thrombasthenia in a young adult: a case report

**DOI:** 10.1097/MS9.0000000000005629

**Published:** 2026-09-02

**Authors:** Manasa Kuppireddy, Dikshitha Bai R

**Affiliations:** Department of Internal Medicine, Manipal Hospital, Bangalore, India

**Keywords:** gingival bleeding, Glanzmann thrombasthenia, platelet, platelet function disorder, aggregation studies, inherited bleeding disorders

## Abstract

**Introduction and importance:**

Glanzmann thrombasthenia is a rare inherited platelet function disorder characterized by defective platelet aggregation and mucocutaneous bleeding. Although it typically presents in childhood, delayed diagnosis in adulthood is uncommon and poses diagnostic challenges, particularly when routine coagulation parameters are normal.

**Case presentation:**

We report a young adult male who presented with recurrent gingival bleeding and severe iron-deficiency anemia without a prior history of childhood bleeding. Routine coagulation studies and platelet counts were normal. Platelet aggregation studies demonstrated absent aggregation to all agonists except ristocetin, confirming the diagnosis of Glanzmann thrombasthenia. The patient was managed with supportive care and transfusion as required.

**Clinical discussion:**

This case highlights the importance of considering inherited platelet function disorders in adults with unexplained mucocutaneous bleeding despite normal initial laboratory investigations. Platelet aggregation studies remain the diagnostic gold standard and are essential to avoid delayed diagnosis and inappropriate management.

**Conclusion:**

Glanzmann thrombasthenia can rarely present in adulthood and should be considered in patients with persistent isolated mucocutaneous bleeding. Early recognition allows appropriate counselling, bleeding prevention strategies, and optimized transfusion planning.

## Introduction

Glanzmann thrombasthenia (GT) is an inherited platelet function disorder caused by qualitative or quantitative defects in the GPIIb/IIIa (αIIbβ3) integrin, which mediates fibrinogen binding and platelet aggregation^[^1–3^]^. Mutations in ITGA2B or ITGB3 result in diminished or dysfunctional receptor expression, impairing primary hemostasis and producing mucocutaneous bleeding of variable severity^[^3–6^]^. Although GT is exceedingly rare globally, with an estimated prevalence of one in a million, it is significantly more common in communities with high rates of consanguinity^[^5–7^]^. Typical manifestations – including epistaxis, purpura, gingival bleeding, and prolonged bleeding after minor trauma – generally appear in infancy or early childhood, and most patients are diagnosed before adolescence^[^5–8^]^.

HIGHLIGHTS

- An adult-onset diagnosis of Glanzmann thrombasthenia without childhood bleeding is rare.
- Persistent isolated gingival bleeding may be the only presenting feature.
- A normal platelet count and coagulation profile can delay diagnosis.
- Platelet aggregation studies are essential for confirming Glanzmann thrombasthenia.
- Early recognition allows for appropriate transfusion planning and prevention of bleeding.

However, delayed recognition in adulthood has been reported when early symptoms are absent, subtle, or misattributed to benign causes^[^6,9^]^. The diagnostic evaluation includes routine hematologic studies, followed by platelet aggregation testing, with light-transmission aggregometry serving as the gold standard^[^6,10^]^. A characteristic pattern of absent aggregation to all agonists except ristocetin strongly supports GT^[^11^]^. This report describes an atypical case of GT presenting in adulthood with no childhood bleeding history.

This manuscript complies with the TITAN 2025 guidelines governing transparency and the responsible use of artificial intelligence in scholarly publishing.

## Case presentation

A 27-year-old South Asian male, born to non-consanguineous parents, presented with a 5-month history of spontaneous gingival bleeding. He denied trauma, epistaxis, hematuria, gastrointestinal bleeding, joint bleeding, or bleeding from other sites. He had not been using antiplatelet or anticoagulant medications. There was no family history of bleeding disorders over three generations.

On examination, he was afebrile with stable vital signs. Conjunctival pallor was prominent. Oral examination showed mild but persistent spontaneous gingival bleeding; the attached gingiva was firm and appeared healthy. Systemic examination revealed no hepatosplenomegaly or other abnormalities. Routine laboratory tests, including blood glucose, glycated hemoglobin, serum electrolytes, renal function, and liver enzymes, were normal. Hemoglobin was critically low at 3 g/dL (reference: 12–15 g/dL). Platelet count was 483 × 10^9^/L (150–400 × 10^9^/L). Peripheral smear demonstrated severe anisopoikilocytosis with microcytic, hypochromic red cells, elliptocytes, teardrop cells, and occasional polychromatophils. Platelets were mildly increased in number. No abnormal cells or parasites were observed, suggesting severe iron-deficiency anemia with reactive thrombocytosis.

Iron studies showed low serum iron with elevated total iron-binding capacity. Coagulation parameters were within normal limits: prothrombin time, 15.5 seconds; activated partial thromboplastin time, 29.2 seconds; and an INR within normal limits. Viral serologies, antinuclear antibodies, and hemoglobin variant analysis were unremarkable. Von Willebrand factor antigen and activity were normal; factor VIII levels were normal; and factor XIII screening was negative (Table 1).

**Table 1:** Significant initial laboratory investigations to affirm a bleeding disorder.

S. No.	Investigations	Result	Biological reference interval	Units
1	Hemoglobin (Hb)	3.0	12–17	g/dL
2	Total leucocyte count (TLC)	5700	4000–11 000	Cmm
3	Platelet count	483	150–400	X109/L
4	Prothrombin time (PT)	15.5	11–16 seconds	–
5	Activated partial prothrombin time (aPTT)	29.2	26–40 seconds	–
6	International normalized ratio (INR)	1.28	–	–
7	Serum iron	7.4	60–180	mcg/dL
8	Total iron binding capacity	512.4	240–400	mcg/dL
9	Viral markers	Negative	–	–

The laboratory findings demonstrated severe iron deficiency anemia with preserved coagulation parameters, prompting further evaluation for an underlying platelet function disorder.

Platelet aggregation studies demonstrated severely reduced aggregation with ADP, arachidonic acid, and collagen, with a normal response to ristocetin, confirming GT (Table 2). Normal vWF and factor VIII levels excluded von Willebrand disease and hemophilia; a normal platelet count made immune thrombocytopenia unlikely (Table 2; Fig. 1).

**Figure 1.**
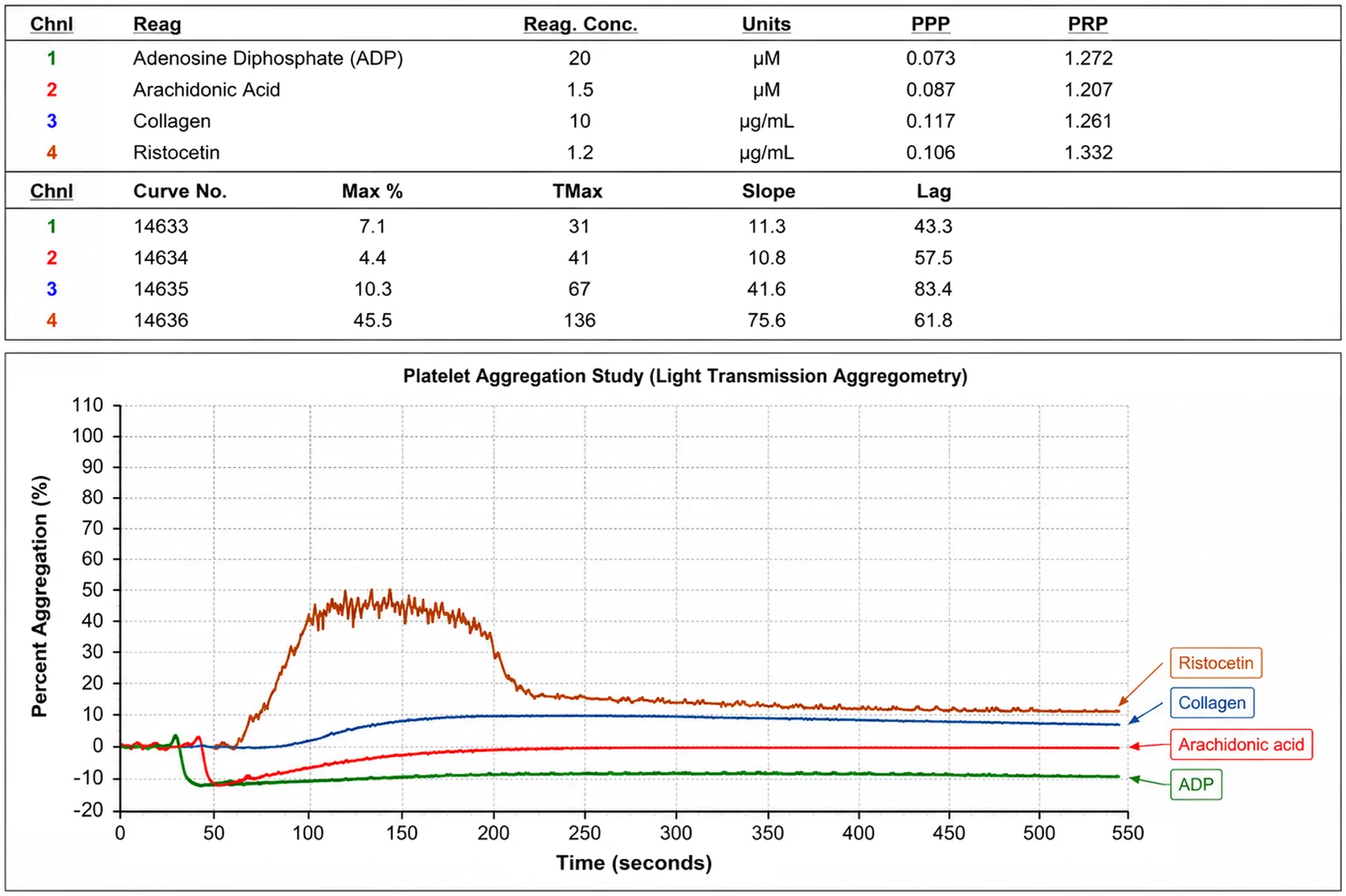
Platelet aggregation study.

Platelet aggregation studies showed markedly reduced responses to ADP, arachidonic acid, and collagen, with relative preservation of ristocetin-induced agglutination, supporting the diagnosis of Glanzmann thrombasthenia.

**Table 2:** Platelet aggregation study.

S. No.	Investigations	Result	Biological reference interval
1	ADP 20 µM	7.1%	50–100
2	Arachidonic acid 1.5 µM	4.4%	50–100
3	Collagen 10 µg/mL	10.3%	50–100
4	Ristocetin – RIPA	45.5%	50–100

The patient received intravenous tranexamic acid (500 mg once daily for 3 days) and two units of packed red blood cells (PRBCs), which raised hemoglobin to 5.2 g/dL. He was discharged with oral tranexamic acid and iron supplementation. Three months later, he returned with worsening gingival bleeding, fatigue, and dyspnea. Hemoglobin was 3.5 g/dL. He received three units of PRBCs, improving hemoglobin to 7.8 g/dL. Following discharge with continued iron therapy, he reported significant improvement at a 1-week follow-up visit, with hemoglobin rising to 10.2 g/dL and only occasional gingival bleeding. The patient was counseled extensively regarding the nature of GT, bleeding risks, avoidance of nonsteroidal anti-inflammatory drugss and trauma, genetic inheritance, and long-term management strategies. Emphasis was placed on meticulous oral hygiene, regular dental care, and the potential for advanced therapies such as hematopoietic stem cell transplantation.

## Discussion

GT is one of the most clearly defined inherited platelet function disorders, yet its diagnosis is frequently delayed when early symptoms are subtle or absent. The disorder results from qualitative or quantitative defects in the GPIIb/IIIa complex, which plays a key role in fibrinogen-mediated platelet aggregation^[^1–3^]^. Despite normal platelet counts and morphology, impaired αIIbβ3 function produces lifelong susceptibility to mucocutaneous bleeding, though clinical severity varies substantially among patients^[^4–7^]^. This heterogeneity contributes to delayed recognition, especially in non-consanguineous populations where clinical suspicion is lower^[^5–7^]^.

Most patients are diagnosed during childhood, with several cohort studies reporting recognition within the first decade of life^[^7–9^]^. However, as illustrated by our case, diagnosis may occasionally be delayed until adulthood, when early bleeding manifestations are absent or attributed to benign conditions such as gingivitis. Comparable cases of late diagnosis have been documented, underscoring the need to include inherited platelet disorders in the differential diagnosis of persistent bleeding in adults^[^9^]^. Delayed diagnosis may lead to cumulative morbidity from repeated mucosal bleeding and anemia, as well as unnecessary treatments or investigations^[^1,6^]^.

The diagnostic evaluation remains challenging. An international survey of laboratories demonstrated substantial variability in diagnostic testing for inherited platelet function disorders and highlighted the challenges associated with limited testing resources and lack of standardization^[^3^]^. Light-transmission aggregometry continues to be the gold standard for GT diagnosis, showing absent aggregation with ADP, epinephrine, collagen, and arachidonic acid while maintaining normal aggregation to ristocetin^[^1,6,11^]^. This characteristic pattern was observed in our patient and strongly supported the diagnosis despite the absence of genetic testing. Although flow cytometry and molecular analysis can provide a definitive diagnosis, their availability is limited in many regions^[^6,12^]^.

The delayed diagnosis in our patient highlights the marked phenotypic variability of GT. Although the disorder typically presents during childhood, rare individuals may remain clinically unrecognized until adulthood despite having an inherited platelet function defect. In our case, retrospective review did not identify any significant childhood bleeding episodes, family history of bleeding disorders, or precipitating factors such as trauma, surgery, infection, or medication use. This atypical presentation emphasizes the importance of considering inherited platelet function disorders even in adults presenting with isolated recurrent mucocutaneous bleeding. Flow cytometric assessment of platelet GPIIb/IIIa expression and molecular genetic testing were not performed because these investigations were not available at our institution. Nevertheless, the characteristic platelet aggregation profile, together with the clinical presentation and exclusion of alternative diagnoses, was considered sufficient to establish the diagnosis.

Management is primarily directed toward controlling acute bleeding and preventing recurrence. Platelet transfusion is widely used for significant or refractory hemorrhage, although repeated exposure poses risks such as alloimmunization to HLA or GPIIb/IIIa antigens, which may reduce treatment efficacy over time^[^1,6^]^. Antifibrinolytics are important adjuncts, particularly for mucosal bleeds, and optimizing dental hygiene is essential for reducing the recurrence of gingival bleeding^[^6^]^. Recombinant activated factor VII (rFVIIa) has also demonstrated efficacy in patients who respond inadequately to platelet transfusions, although access may vary across healthcare settings^[^1,6^]^.

This case illustrates the challenges of diagnosing GT in adulthood, particularly in individuals without a childhood bleeding history or known consanguinity. Persistent or unexplained gingival bleeding should prompt consideration of inherited platelet function disorders. Recognizing the characteristic aggregometry pattern is critical for distinguishing GT from von Willebrand disease, Bernard–Soulier syndrome, and other platelet disorders. Early identification can improve management, guide transfusion strategies, and reduce long-term complications.

## Conclusion

Although GT is typically recognized in childhood, adults may occasionally present with new-onset mucocutaneous bleeding despite having no prior bleeding history. Such atypical presentations may delay diagnosis, as congenital disorders are often overlooked in adult patients. Recognizing the characteristic aggregation pattern is essential for distinguishing GT from other inherited or acquired platelet disorders. Early diagnosis allows appropriate transfusion planning, counseling, and preventive strategies to minimize morbidity.

## Data Availability

The data supporting the findings of this case report are available from the corresponding author.

## Research registration unique identifying number (UIN)

Not applicable to this study.
